# Rab11 Plays an Indispensable Role in the Differentiation and Development of the Indirect Flight Muscles in *Drosophila*

**DOI:** 10.1371/journal.pone.0073305

**Published:** 2013-09-02

**Authors:** Divya Singh, Jagat Kumar Roy

**Affiliations:** Cytogenetics laboratory, Department of Zoology, Banaras Hindu University, Varanasi, India; National Cancer Institute, United States of America

## Abstract

Rab11, an evolutionary conserved, ubiquitously expressed subfamily of small monomeric GTPase has been known to regulate diverse cellular and developmental events, by regulating the exocytotic and transcytotic events inside the cell. Our studies show that Rab11 regulates *Drosophila* adult myogenesis by controlling proliferation and differentiation of the Adult muscle precursors (AMPs). Blocking Rab11 in the AMPs, which fuse to form the Indirect Flight Muscles (IFMs) of fly, renders flies completely flightless and non-viable. The indirect flight musculature, comprising of the differentially patterned dorsal longitudinal muscles (DLMs) and dorsal ventral muscles (DVMs), is affected to different extents. Abrogating or knocking down normal Rab11 function results in severely disrupted IFMs. DLMs forming from larval templates are reduced in number along with a significant reduction in their fibre size. The *de novo* developing DVMs are frequently absent. The DLMs in Rab11 hypomorphs are highly reduced, showing as a small constricted mass in one half of the thorax. Further, Rab11 function is essential for growth of these muscles during later half of adult myogenesis, as down regulation of Rab11 in IFMs results in degenerated muscles and broken fibres. Finally, we show that loss of Rab11 activity in the AMPs result in acquisition of migratory characteristic of myoblast as they show cellular protrusion at their polar ends accompanied with loss of cell-cell contacts. Our data provide the first evidence of a trafficking protein playing an indispensable role in regulating early stages of adult muscle development.

## Introduction

The process of intracellular transport in multicellular organisms mediates a variety of key cellular processes, such as maintenance of homeostasis, development, uptake of nutrients and down-regulation of signal transduction which is regulated by a repertoire of interacting proteins. In eukaryotic cells, several monomeric GTPases, known as Rab proteins, are the major contributors to the process of intracellular transport through vesicles operating between distinct cellular membrane compartments. The process involves cargo selection, vesicle budding, motility, docking and fusion [1,2].

Rab11, an evolutionarily conserved, ubiquitously expressed subfamily of small monomeric GTPase has been known to regulate diverse cellular and developmental events by regulating the exocytotic and transcytotic events in cells. Rab11 localizes to the pericentriolar recycling endosomes, the trans–Golgi network and post-Golgi vesicles [3,4]. A role for Rab11 in proliferation and differentiation of retinal tissues has been demonstrated in Zebrafish [5]. In *Drosophila* Rab11 has been shown to be involved in polarization of oocytes [6,7], post-Golgi trafficking of rhodopsin [8], ommatidal formation, activation of JNK signalling during eye development [9,10] maintenance of germ line stem cells [11], embryonic nervous system development [12] and promoting terminal differentiation of follicle cells in egg chamber [13]. Rab11 has been recently shown to be involved in regulating cell-cell interaction during collective cell migration [14].

The complex developmental process of myogenesis is well conserved from insects to vertebrates and is regulated at different levels by a complex network of proteins. Two distinct sets of muscles are found during *Drosophila* development [15]. The first phase of embryonic myogenesis gives rise to multinucleated larval muscles while the pupal myogenesis gives rise to the adult muscles, which are specifically required for walking and flying. The embryonic and adult myogenesis processes are independent of each other and each involves precisely controlled sequence of events: commitment and specification of muscle precursors, their proliferation and subsequent migration to appropriate target sites, followed by their differentiation giving rise to the final muscle pattern which is accompanied with profound changes in gene expression [16].

Initial studies from our lab have shown that an optimal function of Rab11 is essential for normal myoblast fusion to occur leading to formation of *Drosophila* embryonic muscles [17]. In the present study, we investigated if Rab11 plays a crucial role in the precisely controlled events of adult myoblast proliferation, migration and fusion that are distinctly separated from each other as they occur over a period of several days.

The indirect flight muscles (IFM) of *Drosophila* , constitute more than 70% of the flight muscle mass and are located in the thorax of adult fly. The IFMs are comprised of two groups of muscles: six Dorsal Longitudinal Muscles (DLMs) extending along antero-posterior axis and three sets of Dorsal Ventral Muscles (DVMs) running from dorsal to ventral side of the thorax [18]. The IFMs are seeded by Adult Muscle Precursors (AMPs) that are associated with the presumptive notum region of the wing imaginal disc and are set aside during embryonic myogenesis [18]. These AMPs do not differentiate but undergo extensive proliferation during the larval instars persistently expressing Twist [19,20]. This proliferating myoblast population contain two distinct populations, the Founder Cell (FC) which is chosen from among the group of proliferating AMPs and determines the identity of a specific muscle; the remaining myoblast population is Fusion Competent Myoblasts (FCMs) that fuse to the larval templates to pattern the DLMs and by *de novo* fusion to the founder myoblast, form the DVMs. The fibre size of each muscle is precisely regulated by the number of myoblast fusing which are produced through active proliferation over a prolonged period starting in early larval stage and continuing through the first 24h of pupal myogenesis [21].

To understand the functional requirement of Rab11 during adult muscle development, we chose the large indirect flight muscles of *Drosophila* , because of their large size and highly organized structure with respect to number, position, attachments and innervations.

We observed significant muscle abnormality and flightlessness in flies expressing *UAS-Rab11* ^*N124I*^ (which inhibits the normal Rab11 function by binding to the GTP binding site of the protein) and *UAS-Rab11* ^*RNAi*^ (Rab11 reduction via dsRNA) transgenes. Severe muscle anomalies and subsequent reduced viability in such flies reflects the requirement of Rab11 during adult muscle development. Further, reduced proliferation of AMPs and their inability of establish cell-cell contacts were observed in cells deficient of this trafficking molecule. These results indicate a functional requirement of Rab11 in early stages of myogenesis.

## Material and Methods

### Fly strains

Wild type (Oregon R^+^) and mutant flies were reared at 24^°^C+1^°^C on standard food containing agar, maize powder, yeast and sugar. The following strains were used: *1151-GAL4* (kind gift from Prof. K. VijayRaghavan), *Actin-88F* GAL4 [22], *mhcF3-580GAL4* [22], *UAS-RFP* (Bloomington Stock Center)*, Rab11* ^*mo*^ */TM6B* (generated in lab [9], is a P-insertion allele of Rab11 having an insertion in 5’ regulatory region) *UAS-Rab11* ^*N124I*^ */CyO* and *UAS-Rab11* ^*RNAi*^ [8].

### Lethality assay

The lethality assay was done by counting the total number of embryos from the indicated genotype and placing them on separate agar plates. Embryos developing into larvae, larvae to pupae and finally eclosing as flies were counted separately. The percentage of lethality at each stage of development was calculated between the control and experimental group. Three replicates of each experiment were performed.

### Flight test

Flight test was carried out at room temperature (24^°^C) in the flies 12h after eclosion. For flight testing, pharate pupae were collected and kept in vials. As the flies eclose, they were transferred to empty vials to acclimatize and then each fly was introduced in 1 litre glass cylinder with a centimeter scale fixed on its wall. Flies that could not fly at all and fell straight to the floor of the cylinder were scored as flightless, flies that could reach to the side walls only up to 2 cm of height were scored as having weak flight, those showed wingbeat and could reach to the height of 2 to 10cm were scored as moderate fliers while the ones that showed active wingbeat and strong ability to fly to any distance above 10cm were regarded as good fliers. The weak and moderate fliers were also tested by following the same procedure in a 1 litre glass beaker to provide them sufficient space for flight. The number of flies scored for each genotype was 75 and three replicates of each experiment were performed.

### Muscle preparation

For analyzing the indirect flight muscles in the adults, hemithoraces were frozen in liquid nitrogen, cut sagitally, dehydrated through series of 50%, 70%, 90% and absolute ethanol, cleared in methyl salicylate, mounted in Canada balsam and observed in plane polarized light using a Nikon E800 microscope. 100 hemithoraxes were observed in each case. Three replicates of each experiment were performed.

### Immunofluorescence and acridine orange staining

Wing imaginal disc from the larvae were dissected, fixed in 4% paraformaldehyde for 20min at room temperature (RT), washed in PBT (PBS, 0.3%, Triton X-100) thrice for 10min each, blocked in blocking solution (PBS, 0.1%, Triton X-100, 0.1% FCS and 0.02% thiomersal as antifungal agent) for 2h at RT followed by overnight incubation in the primary antibody in blocking solution at 4^°^C. The tissue were washed thrice in PBT and incubated in secondary antibody for 2h at RT, washed thrice in PBT and mounted in antifadant DABCO (Sigma). The following primary antibodies were used: anti-Rab11 (rabbit, 1:200), anti-twist (rabbit, 1:1000 kindly provided by Maria Leptin, University of Köln), anti-phospho histone (mouse, 1:1000 Milipore). Rabbit secondary antibodies used were Alexa flour 488 conjugated (Molecular probes, USA, 1: 200 dilution), Alexa flour 546 conjugated (Molecular probes, USA, 1: 200 dilution), CY3 conjugated (Sigma, USA, 1: 100 dilution). To assay the extent of apoptosis, the wing imaginal discs from third instar larvae were dissected in PSS, stained with 1µg acridine orange (AO)/ml of PBS for 3 min, washed twice in PBS, mounted in PBS [23,24] and immediately viewed in the Nikon E800 microscope.

### Estimation of myoblast expanse

Imaginal discs from third instar larvae were dissected and processed for immunostaining as described above. The area occupied by the myoblast was calculated as fraction of wing disc area occupied by Twist positive myoblast in control and experimental groups. The density of myoblast was measured by counting the total number of Twist positive myoblast in the notum. For each control or experimental group, wing discs from 21 larvae were sampled randomly.

### Microscopy and Image analysis

For recording the flight behaviour and external morphology of wings, adult flies of the desired genotype were photographed using a Sony Digital Camera (DSC-75) attached to a Zeiss Stemi SV6 stereobinocular microscope. The light/DIC microscopic examinations were carried out with a Nikon E800 microscope with appropriate filter combinations and the images were recorded with a Nikon DXM 1200 digital camera. All GFP or immuno-fluorescence stained preparations were examined with a Zeiss LSM 510 Meta confocal microscope using appropriate lasers, dichroics and filters. The different objectives used for confocal or other microscopy were 10X (0.3NA, Plan Fluor), 20X (0.5NA, Plan, Fluor) or 40X oil (1.4NA, Plan Apo). All the images were assembled using Adobe Photoshop 7.0.

### Statistical Analysis

Sigma Plot 11.0 software was used for statistical analyses. For testing the level of significance between the control and experimental groups, One-Way Annova was performed. 

## Results

### Blocking Rab11 function in the adult flight muscle precursors renders flightlessness and severely affects viability of the flies

In order to comprehend role of Rab11 during adult muscle development in *Drosophila*, the indirect flight muscles (IFMs) of the fly was used as the model for our study. We used the GAL4/UAS system for altering the levels of Rab11 protein and ectopically expressing the dominant negative allele *UAS-Rab11* ^*N124I*^ in the AMPs, which fuse to form the IFMs of the fly. The *1151GAL4* that has been shown to specifically express in the AMPs (Figure S1) was used to drive the expression of transgenes in the adult myoblast. *1151-GAL4* expression starts in the AMPs at the third larval instar stage and continues to express till the complete population of these precursors have fused to form the flight muscles in pupae and later in the adult fibres [25,26].

**Figure 1:**
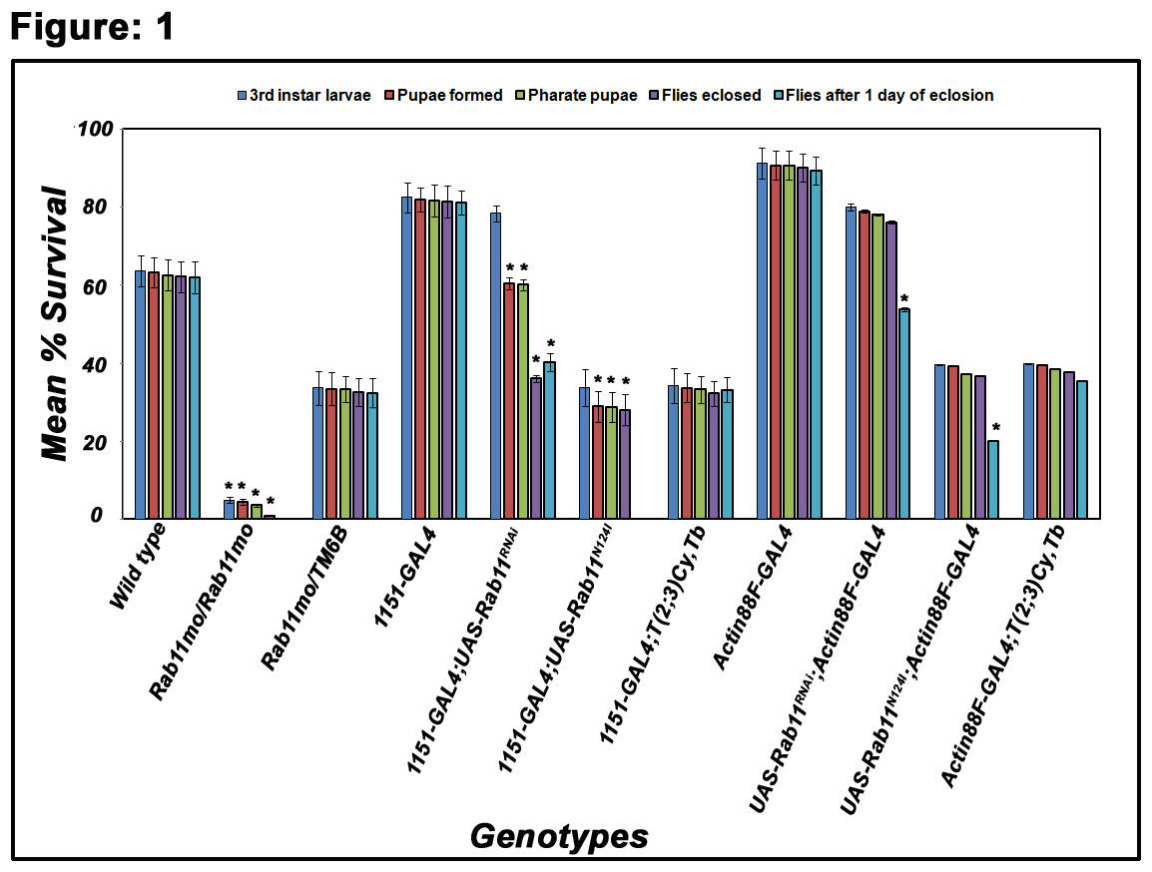
Blocking Rab11 in the adult muscle precursors results in lethality at different stages of development. The vertical bars represent mean (+ S.E) of mean % survivors in three replicates. Percentage of pupae (red bars) and pharate pupae (green bars) were significantly low in *Rab11* ^*mo*^/*Rab11* ^*mo*^, *1151*/*+; UAS-Rab11* ^*N124I*^/*+* and *1151*/*+; Rab11* ^*RNAi*^/*+* as compared to the control. The percentage survival of flies after one day of eclosion (blue bars) showed significant decrease in *1151*/*+; UAS-Rab11* ^*N124I*^/*+* and *1151*/*+; Rab11* ^*RNAi*^/*+*. Asterisk (*) represents significance at p<0.05.

Interestingly, we observed complete flightlessness (Figure 1) in the *1151-GAL4*/+; *UAS-Rab11* ^*N124I*^/+ flies following eclosion. These flies remained stuck to the surface of the food, showed unopened wings and died within few hours of eclosion (Figure 1). To further closely examine this phenotype, the *1151-GAL4*/+; *UAS-Rab11* ^*N124I*^/+ pharate pupae were transferred to an empty vial (with no fly food) preceding eclosion and closely observed for changes during and after eclosion. Intriguingly, the pharate adults kept in the dry environment of the empty vials eclosed normally with fully opened wings but continued to show flightlessness. Also, these flies showed absence of wingbeat, had a poor sense of balance and could not return to their upright position with dorsal side up once they fall (Video S1). To substantiate the flightless phenotype, we performed flight test that demonstrated complete flightlessness (Table 1) in all *1151-GAL4/+; UAS-Rab11* ^*N124I*^ */+* flies observed. The control group showed good flying ability in 77.3% of the flies while 18.6% of flies showed moderate flying (Table 1).

**Table 1:** Flight assay and Wing phenotype.

Genotype	Flight				**Wing Phenotype**
	Flightless	Good	Moderate	Weak	
*Wild type (Oregon R* ^*+*^ *)*	0	77.7+4.3	18.6+1.3	0	Parallel to the body axis
*Rab11* ^*mo*^ */Rab11* ^*mo*^	100	-	-	-	Held Out
*1151GAL4/+*	0	88.43+1.9	11.53+1.9	0	Parallel to the body axis
*1151-GAL4/+; UAS-Rab11* ^*N124I*^ */+*	100	-	-	-	Held Out
*1151-GAL4/+;UAS-Rab11* ^*RNAi*^ */+*	60.8+6.2	-	-	39.1+6.2	Held Out
*Actin88F GAL4/+*	0	89.73+1.9	10.1+1.8	0	Parallel to the body axis
*Actin88F GAL4/+;UAS-Rab11* ^*N124I*^ */+*	100	-	-	-	Held Out
*Actin88F GAL4/ +;UAS-Rab11* ^*RNAi*^ */+*	99.7+0.2	-	-	0.8+0.8	Held Out

In order to further comprehend, if there is an absolute requirement of Rab11 protein during formation of the IFMs, we used *UAS-Rab11* ^*RNAi*^ to knock down Rab11 specifically in the myoblast, and checked if this had any effect on flight and viability of the flies. We observed that a significant (29.3%) of *1151-GAL4/+*; UAS*-Rab11* ^*RNAi*^ */+* progenies could not eclose and died as pharate adults confirming a pharate adult lethality (Figure 1). On closely examining these flies, we found that the pharate adult struggle to move out of the puparium case and only 44% of them were able to eclose (escapers) while the rest died. 60.8% of the escaper flies showed inability to fly, and the rest 29% were weak flyers (Table 1).

Since, the hypomorphic allele of *Rab11* (*Rab11* ^*mo*^ */TM6B*) is known to have reduced protein, owning to a P-element insertion in the 5’ regulatory region of the *Rab11* gene and reduced viability [9], we investigated these flies for any such muscle defects. Interestingly, we observed that *Rab11* ^*mo*^ */TM6B* produced significantly low (1.2%) of homozygous *Rab11* ^*mo*^ viable adults (Figure 1) in contrast to the expected 25%. The lethality was observed to occur at various larval and pupal stages of development. The viable flies showed complete inability to fly and subsequently died (Figure 1, Table 1).

**Figure 2:**
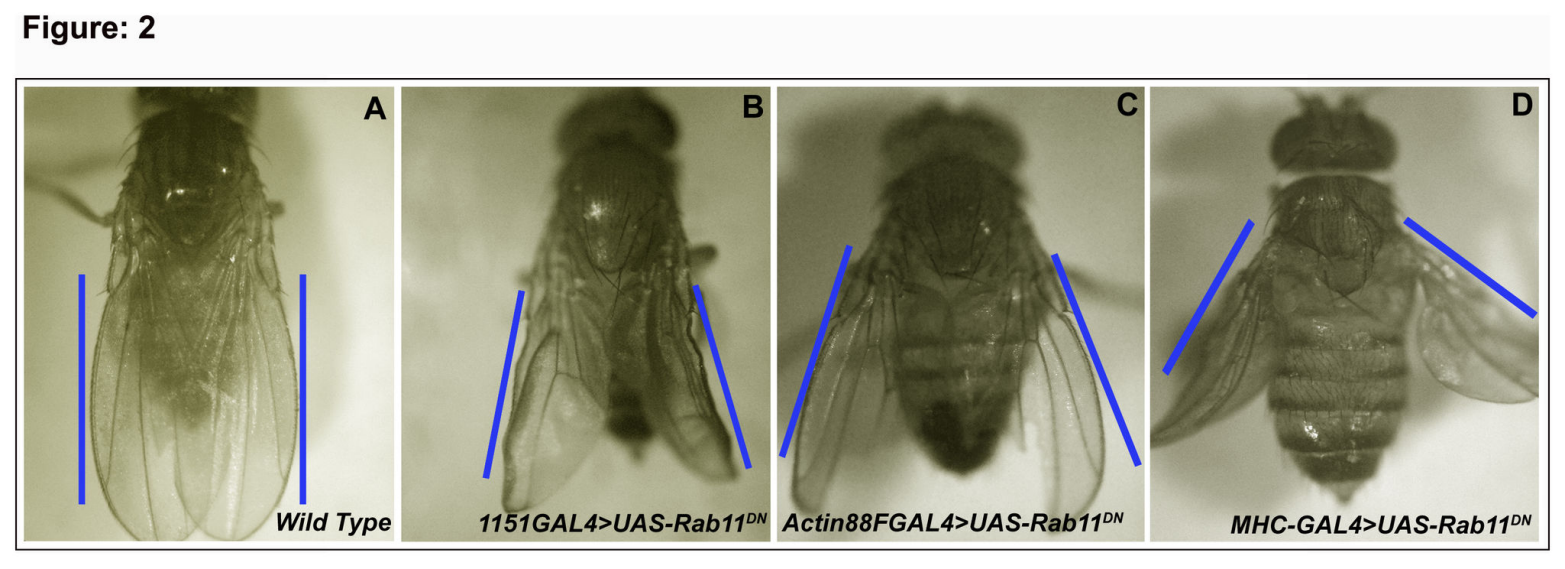
Wing position phenotype in adult flies on expressing dominant negative Rab11 and *Rab11* ^*RNAi*^ (A) Normal wing position observed in a Wild type fly (B) Abnormal wing position phenotype (wing held out at different angles to the axis) on overexpression of dominant negative Rab11 using the *1151GAL4* driver (C) *UAS-Rab11* ^*N124I*^/*+; Actin88F*-GAL4/+ flies showing held out wings (D) *mhc-GAL4> UAS-Rab11* ^*N124I*^ flies with wings held out at almost 45^°^ angle to the axis.

Also, both the *1151-GAL4/+; UAS-Rab11* ^*N124I*^ */+* and *1151-GAL4/+*; UAS*-Rab11* ^*RNAi*^ */+* flies showed an held-out wing position phenotype, in which the wings are held out at different angles from the body axis instead of being parallel to it (Figure 2A, B). The held-out wing phenotype was 100% penetrant in these flies but the expressivity of the phenotype varied as the wings were held out at various angles to the body axis.

From these above phenotypic observations, we had a clear indication of Rab11 playing imperative role in attributing flight ability to *Drosophila* by participating in the flight muscle development which in turn affects the viability of the flies.

### Rab11 is required for the differentiation and de-novo development of dorsal longitudinal and dorsal ventral muscles

**Figure 3:**
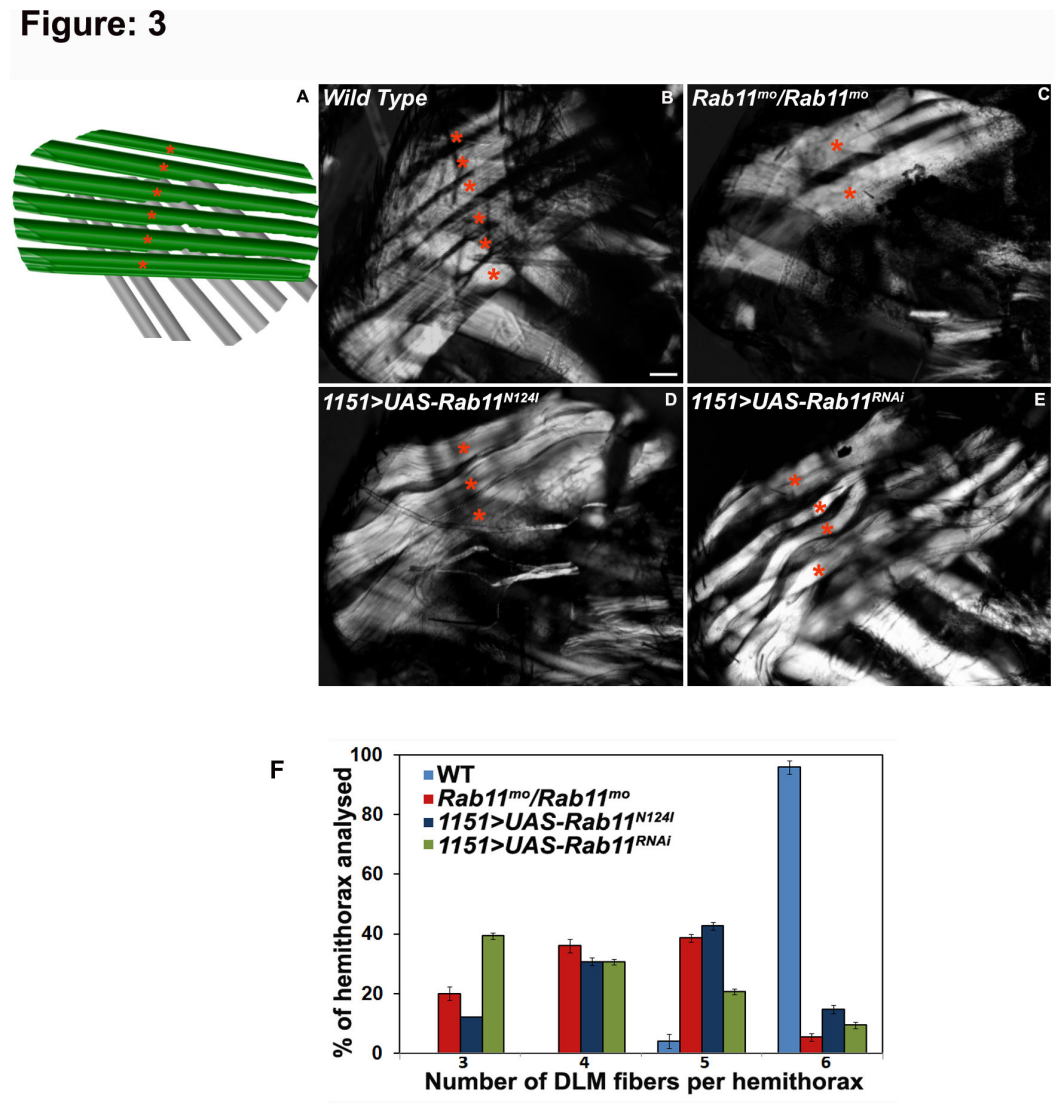
Inhibiting Rab11 function in AMPs prevents differentiation of the dorsal longitudinal muscles. (A) Schematic showing hemithorax of an adult *Drosophila* with six dorsal longitudinal muscles (DLMs in green) indicated by red asterisks. Anterior is to the left and dorsal is to the top in this and all subsequent images. (B) Thoracic preparation of a hemithorax from a wild type fly showing six, well differentiated DLMs marked with asterisks in red; (C) *Rab11* ^*mo*^ */TM6B* homozygotes showing undifferentiated and reduced DLMs. The DLMs in *Rab11* ^*mo*^ homozygotes can be observed as compact structures occupying only half of the thorax as compared to their wild type control; (D) In *1151*/*+; UAS-Rab11* ^*N124I*^/*+* thoraces, three DLM fibres can be observed, showing complete failure of differentiation as the larval templates fail to split into adult fibres (E) *1151*/*+*; UAS*-Rab11* ^*RNAi*^/*+*fly thoraces, occasionally show four or five DLM fibres that are thinner as compared to the controls and are often present as ‘wavy’ structures, (F) Graph shows the number of DLM fibres per hemithorax in different genotypes. Presence of all six DLMs are observed in very few hemithoraces of Rab11 mutant, *1151*/*+; UAS-Rab11* ^*N124I*^/*+* and *1151*/*+*; UAS*-Rab11* ^*RNAi*^/*+*, compared to 96% control hemithoraces with intact six DLMs. Wildtype in blue bars, *Rab11* ^*mo*^/*Rab11* ^mo^ in red bars, *1151*/*+; UAS-Rab11* ^*N124I*^/*+* in navy blue and *1151*/*+*; UAS*-Rab11* ^*RNAi*^/*+* in green bars. Scale bar represent 20 µm.

The flight ability of *Drosophila* is attributed to the indirect flight muscles (IFMs) which are the major bulk of muscles located in the thorax. The DVMs that work as ‘wing elevators’ are present as three individual bundles, each consisting of three, two and two fibres, respectively, while the six dorsal longitudinal muscles (DLMs), also known as ‘wing depressors, run from the anterior to the posterior of each hemithorax (Figure 3A). Through the combinatorial action of these DLMs and the DVMs the fly attains flight. To determine if the flightlessness and wing position defects in *Rab11* ^*mo*^ */TM6B*, *1151-GAL4/+; UAS-Rab11* ^*N124I*^ */+* and *1151-GAL4/+*; UAS*-Rab11* ^*RNAi*^ */+* flies, were due to anomalies in their flight muscle architecture, we examined the thoracic musculature in these flies, using plane polarized optics. Interestingly, substantial disorganization and several anomalies in the IFM musculature of these flies were observed. Thoraces in *Rab11* ^*mo*^ */TM6B* adults, showed intriguingly reduced DLMs, occasionally showing as three thin fibres occupying only half of the thoracic space (Figure 3C) in comparison to the wild type controls where the DLMs span the whole thorax in longitudinal direction (Figure 3B) and had six thick DLM fibres running across the thorax from anterior to posterior ends (indicated by asterisks in Figure 3A, B). On expressing the dominant negative Rab11 protein in the AMPs in *1151-GAL4/+; UAS-Rab11* ^*N124I*^ */+*individuals, we observed significant reduction in the number of DLM fibres in each of the thoraces. Majority of thoraces in *1151-GAL4/+; UAS-Rab11* ^*N124I*^ */+* flies showed either three (39.3%) or four (30.6%) DLMs compared to the six fibres (96%) observed in wild type controls (Fig. 3D F). To check if knocking down Rab11 in the adult muscle precursors in *1151-GAL4/+; UAS-Rab11* ^*RNAi*^ */+* flies, showed similar muscle abnormalities, we observed the thoracic preparations of these flies and found considerable loss of muscle structure along with absence of correct number of DLMs. The DLM fibres in these thoraces appeared as ‘wiggly’ structures that were occasionally disconnected to the cuticle at the two ends of the thorax (Figure 3E). Instead of presence of all six DLM fibres, we occasionally observed the remnant of first and the third larval templates in these thoraces that account for the reduced number of DLMs observed in this case (Figure 3E, F).

**Figure 4:**
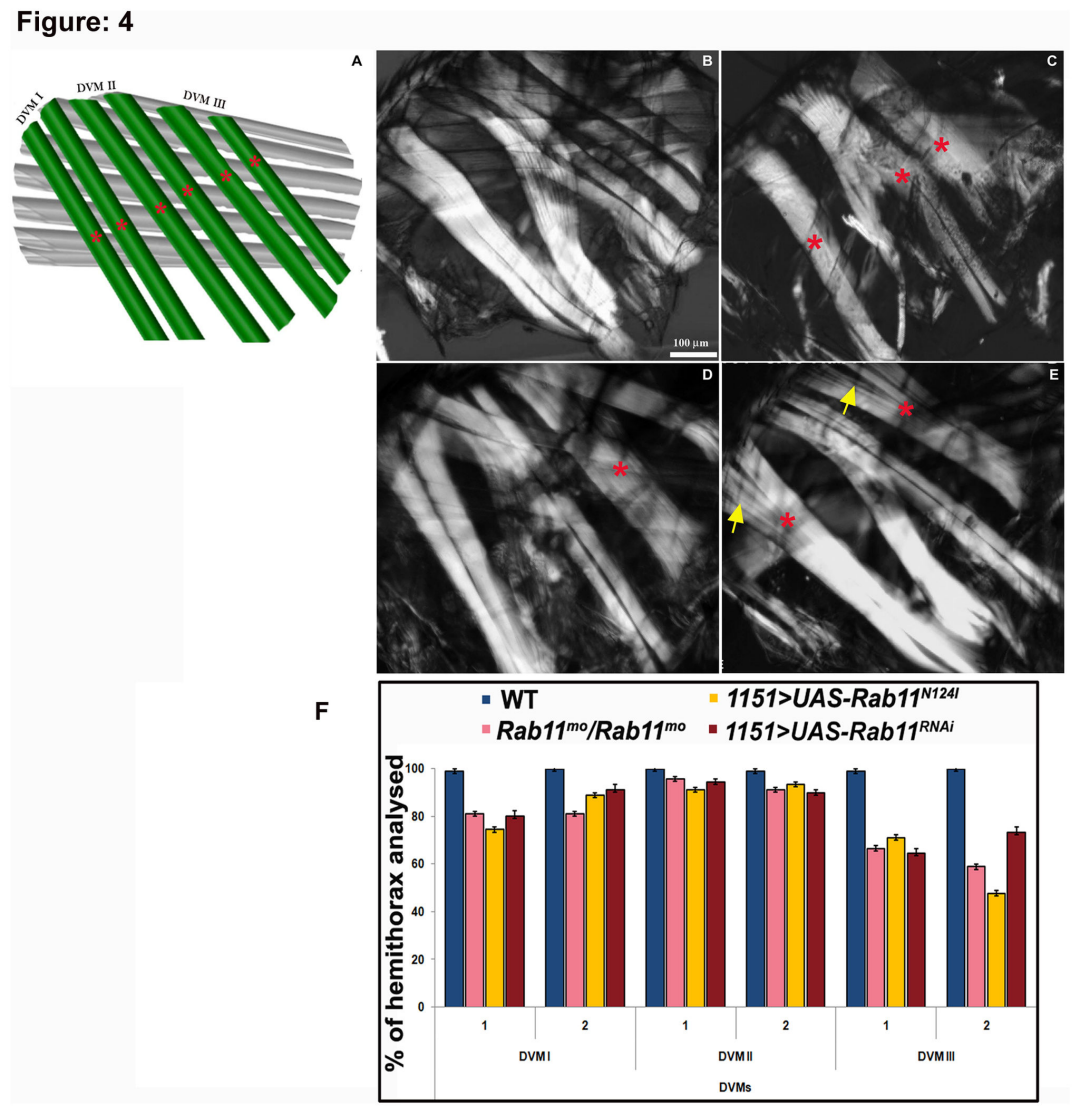
*de novo* formation of individual DVM fiber fails to occur in the absence of Rab11 (A) Schematic showing hemithorax of an adult *Drosophila* containing dorsal ventral muscles that are present as three individual bundles consisting of three, two and two muscles each indicated by red asterisks. The third muscle of the DVM I is usually not visible as it lies in a different plane. Anterior is to the left and dorsal is to the top in this and all subsequent images. (B) Wild type hemithorax showing DVM I, II and III marked with asterisk in yellow (C) *Rab11* ^*mo*^ */TM6B* homozygotes showing presence of a single DVM I and DVM II muscle fibre instead of two muscles observed in the control thoraces. The DVMs also appear to be noticeably thin. (D) *1151*/*+; UAS-Rab11* ^*N124I*^/+ hemithorax showing complete absence of the second muscle of DVM III. Single muscle in DVM III bundle has been marked with red asterisk (E) *1151*/*+*; UAS*-Rab11* ^*RNAi*^/*+* hemithoraces showing single muscle fibre in DVM I and DVM III bundle. Inter-filament spaces present between fibres are marked with yellow arrows. Scale bar represent 100 µm (F) Summary showing the effect on fibre number by comparing the number of fibres present in each hemithorax of an adult fly. Wildtype in blue bars, Rab11^mo^/Rab11^mo^ shown in pink bars, *1151*/*+; UAS-Rab11* ^*N124I*^/*+* in yellow and *1151*/*+*; UAS*-Rab11* ^*RNAi*^/*+*in red. Standard error bars are shown for each of genotypes.

Since, the second set of indirect flight muscles, the DVMs, utilize a completely distinct mode of patterning, we followed up these DVM structures, to know if Rab11 has a functional requirement in configuring these developmentally distinct muscles as well. The DVMs that appear in three sets; DVM I consisting of 3 fibres, DVM II and DVM III with 2 fibres each, are formed using the dumbfounder positive founder cells. The effect of compromised Rab11 function on DVM fibres was quantified by determining the number of fibres in each of the three DVM bundles. Individual muscle fibre from each of these DVM bundles was absent in majority of thoraces. The third DVM seems to be most severely affected with only 47.7% of the fly thoraces showing the presence of both fibres of the bundle in *1151/+; UAS-Rab11* ^*N124I*^ */+* while, the second fibre of the bundle was absent in 52.3% of the fly thoraces (Figure 4D, F). DVM I also showed reduced muscles as 25.6% of the flies in *1151/+; UAS-Rab11* ^*N124I*^ */+* and 20% in *1151GAL4/+; UAS-Rab11* ^*RNAi*^ showed absence of both fibres of the bundle (Figure 4D, F). The *Rab11* ^*mo*^ hypomorphs showed DVMs that were drastically reduced in mass along with absence of individual DVM fibres giving the appearance of an empty thoracic space (Figure 4C). The *1151-GAL4/+; UAS-Rab11* ^*RNAi*^ thoraces showed interfilament spaces between the muscle fibres with loose myotubes contact (Figure 4E).

**Figure 5:**
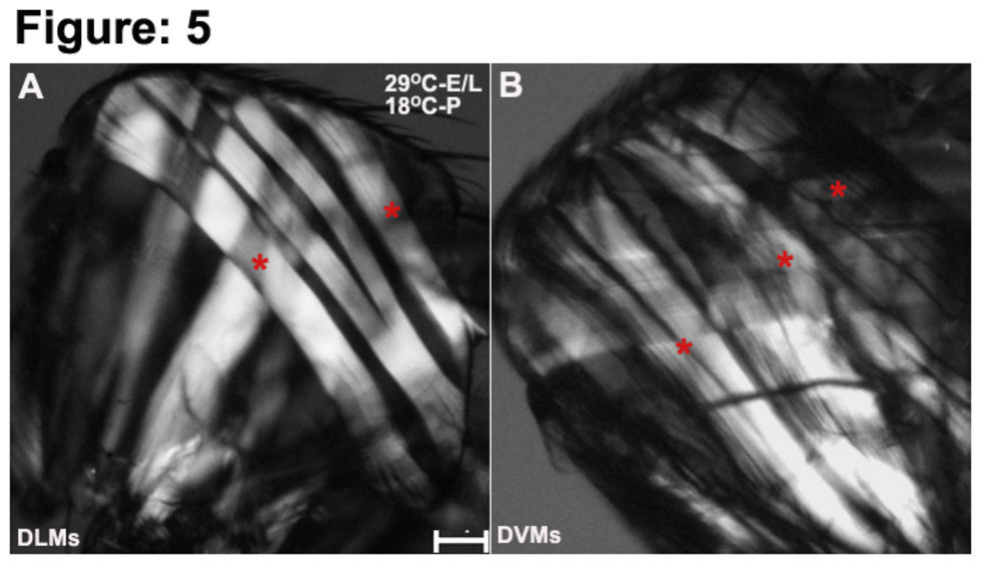
Rab11 function in the adult muscle precursors during early adult myogenesis is crucial for IFM development. (A) When progenies of the genotype *1151-GAL4; UAS-Rab11* ^*N124I*^ *; tubGAL80* ^*ts*^ were raised at 29.5^°^C during the embryonic and larval stages and then moved to 18^°^C for the pupal phase, the DLMs were reduced in number and showed absence of splitting; (B) individual DVMs fibres were reduced in number in *1151-GAL4; UAS-Rab11* ^*N124I*^ *; tubGAL80* ^*ts*^ progenies.

In order to determine if the muscle phenotype observed in *1151-GAL4/+; UAS-Rab11* ^*RNAi*^ and *1151-GAL4/+; UAS-Rab11* ^*N124I*^ genotypes is due to an absolute requirement of Rab11 in the myoblast during larval stages, we used the Gal80^ts^ system to allow a temporal regulation of transgene expression [27]. Gal80 protein represses the GAL4 once it is bound to it. Gal80^ts^ is a temperature-sensitive mutation and inactivates the repressor function of Gal80 at restrictive temperatures (29^°^C) while at permissive temperature the GAL80 binds to GAL4 and blocks the activation of UAS-transgene. The restrictive and permissive temperatures in our experiments were 29.5^°^C and 18^°^C, respectively. The embryos and larvae of *1151-GAL4; UAS-Rab11* ^*N124I*^ *; tubGAL80* ^*ts*^ were grown at 29.5^°^C and shifted to 18^°^C at 0h APF.The adult thoraces showed reduced number of DLMs and poorly developed DVMs (Figure 5A, B) while the converse resulted in a normal IFM muscle architecture. These results show an absolute requirement of Rab11 during *Drosophila* IFMs development, with an important role to play during the differentiation of DLMs, as well as during the *de novo* formation of DVMs.

### Alteration of Rab11 in the indirect flight muscles allows differentiation but subsequently induces degeneration

The adult myogenesis is broadly divided into two phases: differentiation and growth. During early stages of adult muscle development the muscle precursors actively proliferate and fuse to the larval templates to pattern the DLMs and by *de novo* fusion give rise to the DVMs. This process of muscle diffrentiaion is complete by 24APF and is immediately followed by the growth phase that continues till all of the myoblasts have fused to the myotubes. Since both these phases must occur for proper muscle development, we hypothesized a functional requirement of Rab11 during later stages of muscle development as well. To test this hypothesis we allowed active myoblast proliferation and subsequent differentiation of the DLMs and DVMs to advance in the presence of functional Rab11 but restricted Rab11 function by using *Actin88F* driver during the muscle growth phase, in which the muscles increase in size by the continuous process of myoblast fusing to the muscle fibers. The *Actin88F* is a IFM specific actin isoform and the driver mimic the IFM expression of Act88F protein with the expression first detected at around 40h APF, soon after the differentiation of the IFMs is completed [22].

**Figure 6:**
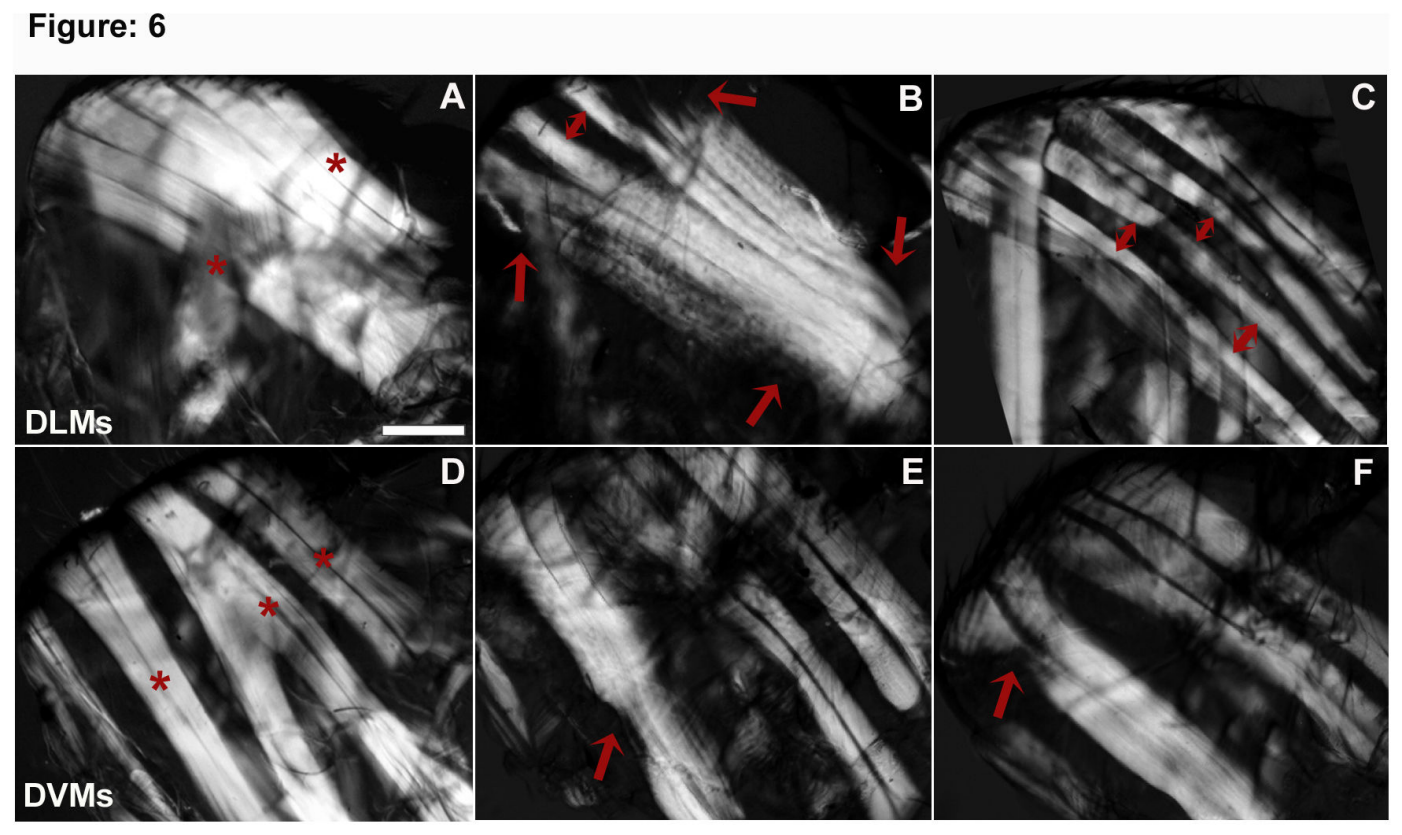
Depletion of Rab11 in the indirect flight muscles during their period of growth leads to their degeneration and thinning. (A) to (F) show representative examples of the DLM and DVM muscle phenotypes in Rab11 altered conditions; (A) *Actin88F-GAL4* flies showing well arranged DLMs, where the first and the sixth DLMs are marked with the red astriek; (B) *UAS-Rab11* ^*N124I*^/*+; Actin88F*-GAL4/+ hemithoraces showing broken and degenerated muscle ends, indicated by red arrows. The DLMs give a “see through” appearance due to degeneration of the muscles ultimately resulting in muscle thinning. The DLMs occasionally show abnormal large gaps between two consecutive fibres, indicated by double headed arrows. These gaps are completely absent in the controls muscles (C) *UAS-Rab11* ^*RNAi*^/*+; Actin88F-GAL4* /+ hemithoraces show significantly thin and abnormally spaced DLMs (D) *Actin88F-GAL4* fly hemithoraces showing DVM I,II and III, marked red astrieks (E) and (F) *UAS-Rab11* ^*N124I*^/*+; Actin88F*-GAL4/+ and *UAS-Rab11* ^*RNAi*^/*+; Actin88F*-GAL4/+ hemithoraces showing degenerated DVMs marked by red arrows. Scale bar represent 100 µm.

Remarkably, both the *UAS-Rab11* ^*N124I*^ */+; Actin88F*-GAL4/+ and *UAS-Rab11* ^*RNAi*^ */+; Actin88F*-GAL4/+ flies, showed significant improvement in viability at all stages of development and eclosion (Figure 1). These flies can also be maintained in viable state. However, while looking at the IFM in these hemithoraces, we observed significant degeneration and thinning, with some muscles appearing to completely degenerate at their ends. The DLMs in some of the hemithoraces were thin and gave a ‘seethrough’ appearance (Figure 6B). The *UAS-Rab11* ^*RNAi*^ */+; Actin88F*-GAL4/+ hemithoraces showed extreme retardation of DLM fibre growth, to an extent that DLMs appeared as thread like structure with large spaces between two neighbouring DLMs, contrary to the closely apposed DLMs present in the controls (Figure 6C). More interestingly, the number of DLMs in these thoraces was comparable to the control, ruling out possible splitting defects and indicating normal muscle differentiation preceding the growth. The DVMs on the other hand were less severely affected but occasionally showed degenerated fibres as well (Fig. 6E F).

To further verify that these effects are due to altered Rab11 function alone, we used *mhcF3580-GAL4*, recombined with RFP, which first shows its expression at about 40h APF. Supporting the above data, the *mhcF3-580GAL4*/*UAS-Rab11* ^*N124I*^ flies showed reduced RFP expression in their IFMs in comparison to the intense RFP expressing IFMs of the *mhcF3-580GAL4* control flies. The DLMs and the DVMs in the *mhcF3-580GAL4*/*UAS-Rab11* ^*N124I*^ and *mhcF3-580GAL4/UAS-Rab11* ^*RNAi*^ were poorly developed (Figure S2). We also observed defects in adult muscles other than IFMs, where *mhcF3-580GAL4* has a specific expression. Together, these data suggest roles for Rab11 in both early and late stages of adult muscle development.

### Rab11 is required during proliferation of the indirect flight muscle precursors

**Figure 7:**
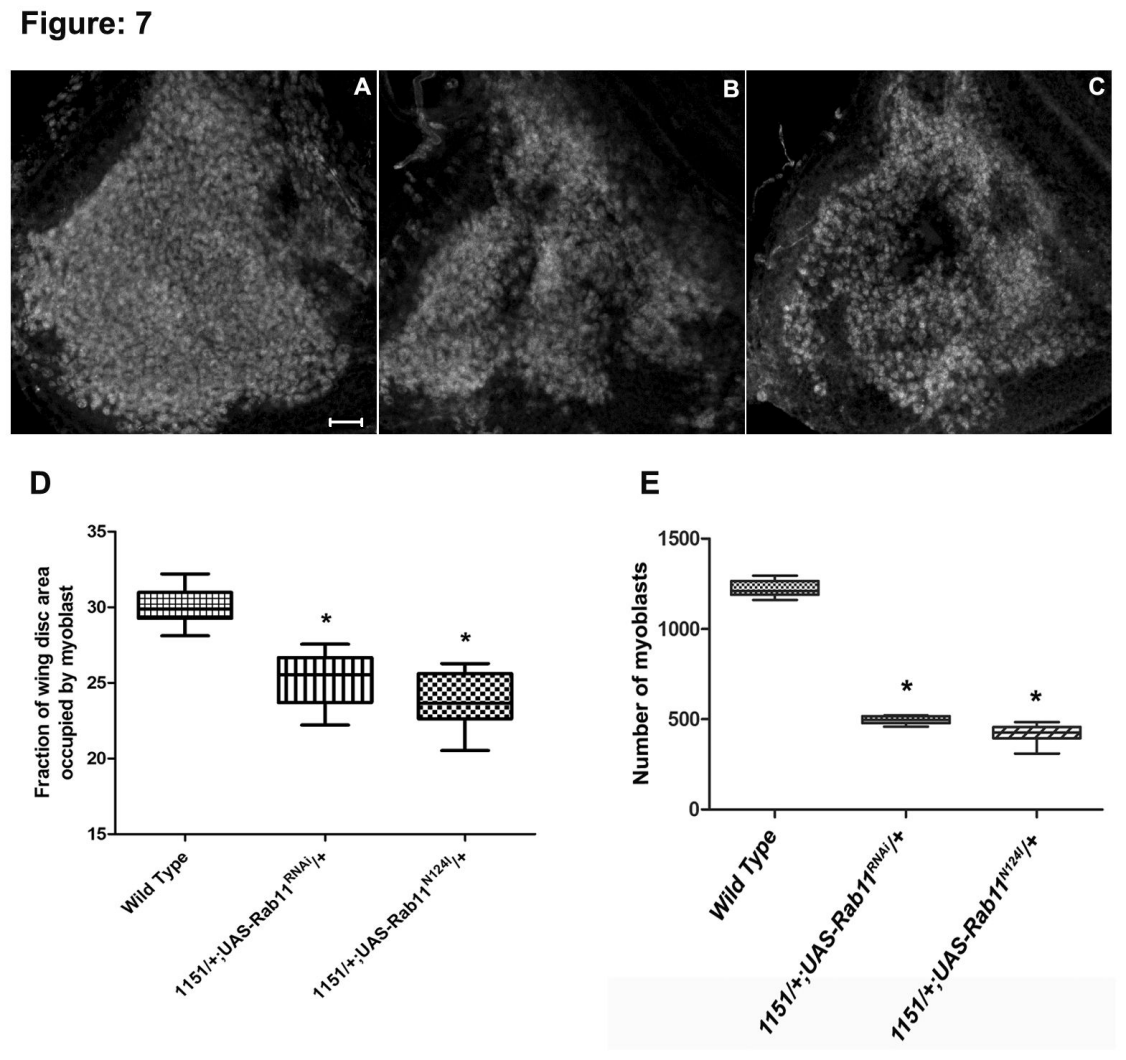
Loss of Rab11 reduces the myoblast expanse and notum size of the wing imaginal disc. (A) Wild type wing imaginal disc notum showing adult muscle precursors; (B) *1151*/*+; UAS-Rab11* ^*N124I*^/*+* and (C) *1151*/*+*; UAS*-Rab11* ^*RNAi*^/*+*; (F) The area occupied by the myoblast in the imaginal disc of both *1151*/*+; UAS-Rab11* ^*N124I*^/*+* and *1151*/*+*; UAS*-Rab11* ^*RNAi*^/*+* is significantly reduced. (E) In addition the density occupied by the myoblst is also reduced. ***p< 0.001 in both cases. Scale bar represent 20 µm.

The DLMs and the DVMs are formed by myoblast that are derived from the wing disc of the third instar larva [28]. To test whether the reduction in muscle number and fibre size was a consequence of transformed myoblast pool that fuse to give rise to the DLMs and DVMs, we examined the myoblast population in the notum of the wing imaginal disc by labelling it with a myoblast specific marker, Twist. The density and area of the myoblast was compared between the control and *1151/+; UAS-Rab11* ^*N124I*^ */+* wing disc. The myoblast expanse was calculated as the fraction of wing disc area occupied by twist positive myoblast. The myoblast occupied 23% of the total area of the wing disc in the *1151/+; UAS-Rab11* ^*N124I*^ */+* larvae (Figure 7D) as compared to the 30% in controls, which was significantly different (P<0.0001). In Rab11 knockdown *1151GAL4/+; UAS-Rab11* ^*RNAi*^ wing disc, the area occupied by myoblast was approximately 25% of the total wing disc area, which was again significantly different (P<0.0001) from the controls (Figure 7C). No significant difference was observed between the myoblast in *1151/+; UAS-Rab11* ^*N124I*^ */+* and *1151GAL4/+; UAS-Rab11* ^*RNAi*^ *.* We also observed a slight decrease in the size of the notum in *1151/+; UAS-Rab11* ^*N124I*^ */+* and *1151GAL4/+; UAS-Rab11* ^*RNAi*^ animals which may be a direct consequence of reduction in the myoblast expanse. However, there was no change in the overall size of the wing imaginal disc in the above genotypes. The density of myoblast was measured by counting the total number of myoblast in the notum and we observed a decrease in the number of AMPs. A reduction of 41.6% was observed in the number of AMPs in *1151GAL4/+; UAS-Rab11* ^*RNAi*^ while 37.5% reduction in the density was observed in *1151/+; UAS-Rab11* ^*N124I*^ */+*. Reduction in the myoblast expanse and density suggested a probable role of Rab11, in regulating myoblast number either by regulating their proliferation or by inducing cell death. To check for the apoptosis occurring in *1151-GAL4/+; UAS-Rab11* ^*N124I*^ */+* and *1151-GAL4/+; UAS-Rab11* ^*RNAi*^ myoblast, we used acridine orange staining to observe the apoptotic cells and found no significant differences in the number of dead cells between the control, *1151/+; UAS-Rab11* ^*N124I*^ */+* and *1151GAL4/+; UAS-Rab11* ^*RNAi*^, suggesting that the reduced myoblast expanse was not due to cell death (Figure S3).

**Figure 8:**
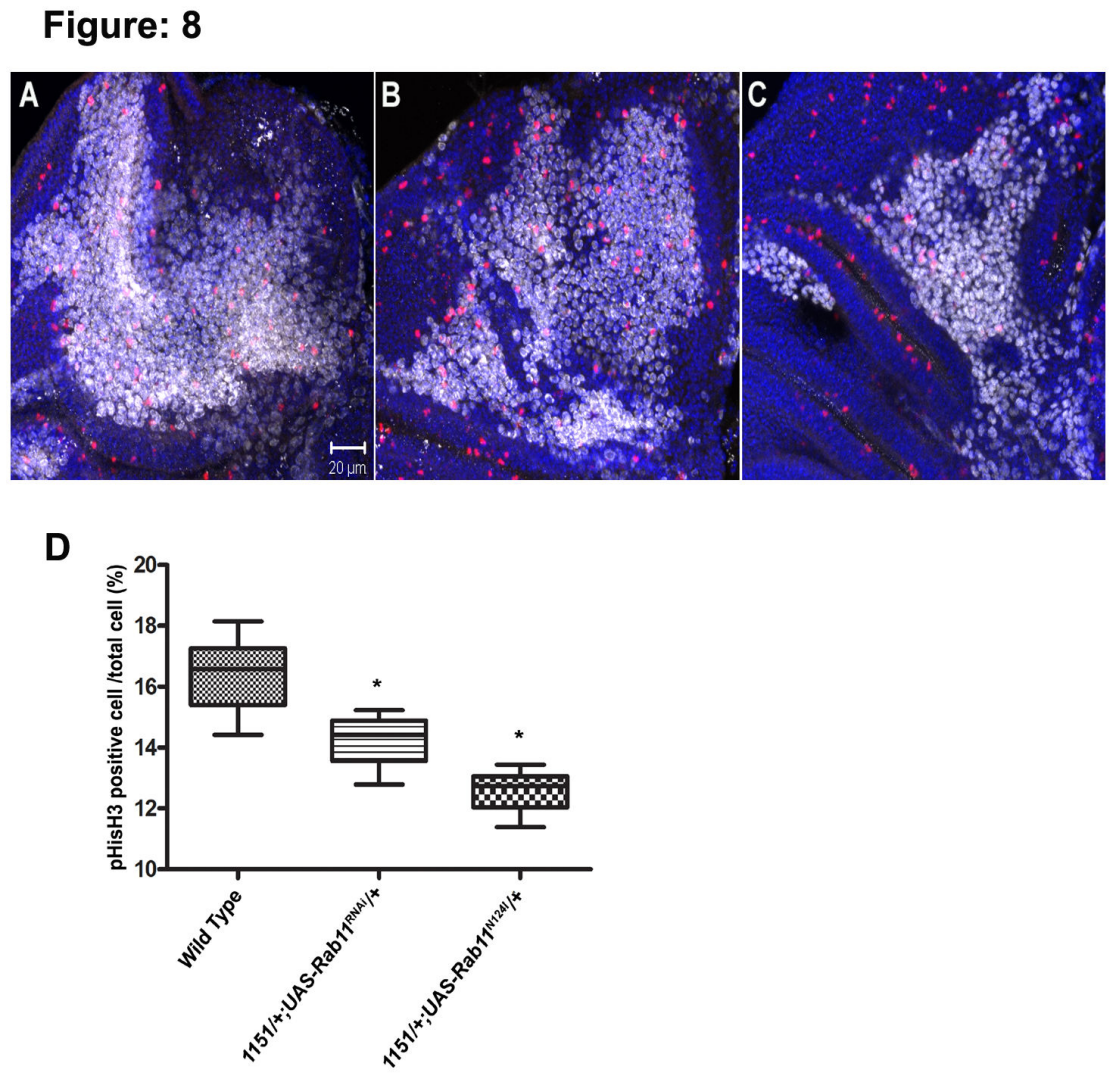
Impaired proliferation of the adult muscle progenitors in Rab11 altered condition Mitotic AMP population in the wing disc notum were immunostained with anti-phospho-histone H3 antibody. The twist positive myoblast (white) cross labelled with anti-phospho-histone (red) and DAPI in blue (A) *1151 GAL4* control (B) *1151*/*+; UAS-Rab11* ^*DN*^/*+* and (C) *1151*/*+*; UAS*-Rab11* ^*RNAi*^/*+* wing disc. The total number of Mitotic phase cells in a representative area of 4500 µm^2^ were counted during the period of active proliferation of myoblast. (D) The number of myoblast positive for phospho-histoneH3 are significantly lower in both *1151*/*+; UAS-Rab11* ^*N124I*^/*+*, *1151*/*+*; UAS*-Rab11* ^*RNAi*^/*+* than control. ***p< 0.001 by t-test. Error bars indicate standard error.

Further, we examined the proliferating myoblast by immunostaining for an M-phase marker, phospho-Histone H3 (pHisH3) that labels the cells in M-phase. The proliferation rates of the myoblast were measured as the fraction of Twist positive myoblast that also labelled with pHisH3. Since the myoblast actively proliferate through the third larval instar, we checked for the proliferating cells in the notum of the wing disc. Interestingly, the wing disc notum from *1151-GAL4/+; UAS-Rab11* ^*N124I*^ */+* and *1151-GAL4/+; UAS-Rab11* ^*RNAi*^ larvae showed reduced number of twist labelled pHisH3 positive cells in comparison to controls (Figure 8A–C) and the difference was found to be statistically significant (p<0.001) in both the cases (Figure 8D). This demonstrated an absolute requirement of Rab11 activity in proliferation of adult muscle precursors.

### Altering Rab11 in muscle precursors results into changes in myoblast morphology and loss of cell adhesion

**Figure 9:**
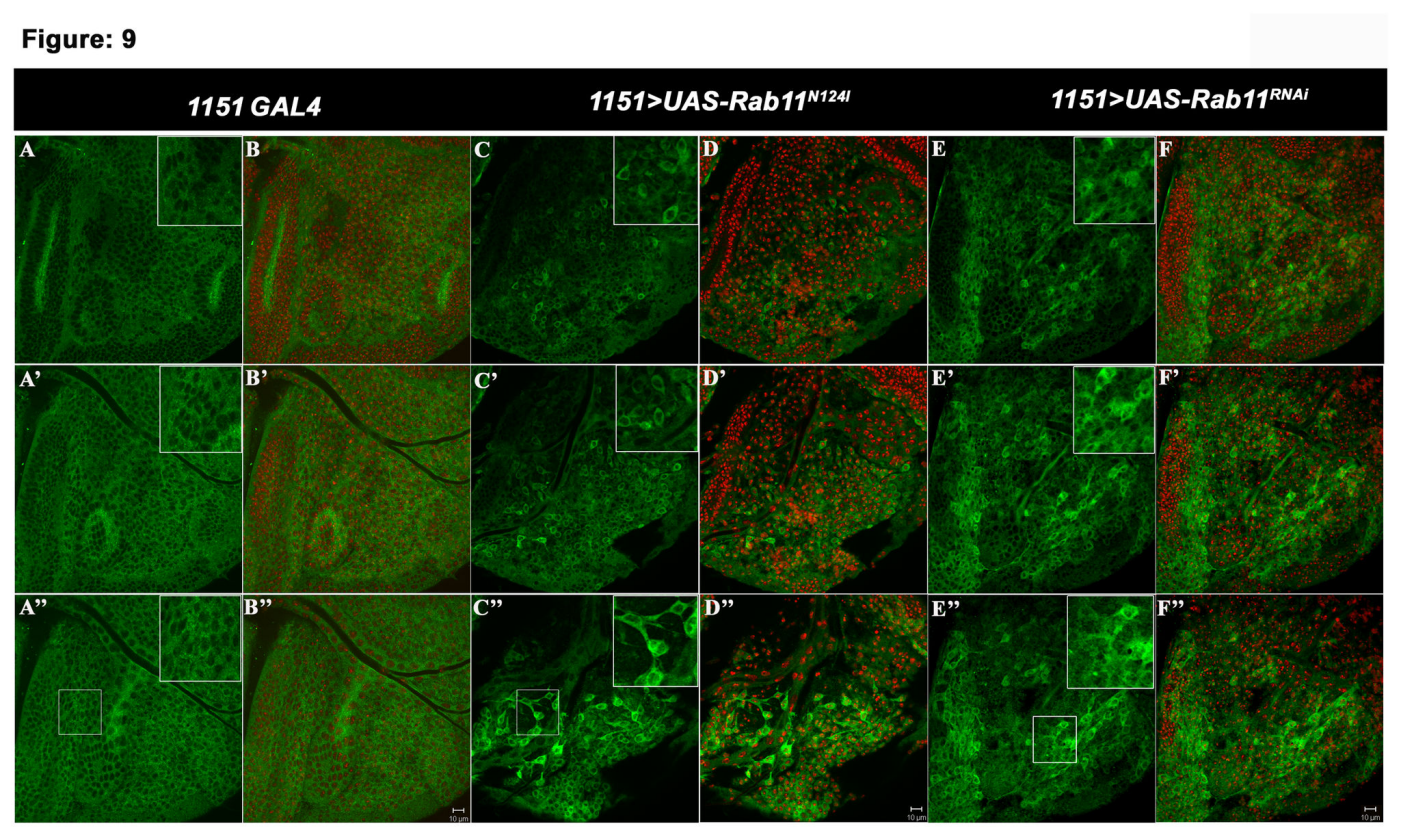
Rab11 is mislocalized in the AMPs resulting in altered morphology and loss of cell-cell contacts. Optical line sections of wing imaginal disc notum at an interval of 1µm, harbouring the AMPs. The sections are displayed in the order of basal to apical polarity of the disc; (A-A’’’) *1151-GAL4/+* notum showing punctuate localization of Rab11 in green; (B-B’’’) merged image showing DAPI in red and Rab11 in green; (C–C’) Ectopic expression of *UAS-Rab11* ^*N124I*^ shows complete loss of punctuate localization of Rab11 in *1151*/*+; UAS-Rab11* ^*DN*^/*+* AMPs. Instead the protein is localized at the apical ends of the AMPs that show ‘tear drop’ morphology; (C’’’) Myoblast in the basal layers are transformed to spindle like cells and show complete absence of cell-cell contacts; (D-D’’’) merged image; (E-E’’’) *1151*/*+; UAS-Rab11* ^*RNAi*^/*+* wing disc notum showing completely disorganized AMPs population and loss of Rab11 punctuate localization. Scale bar represents 10µm.

To define the requirement of Rab11 during adult muscle precursor differentiation, we studied the subcellular distribution of Rab11 in the AMPs using anti-Rab11 antibody. Rab11 exhibited a punctuate localization in the control adult muscle precursors harbouring the larval wing disc (Figure 9A–A’’). We checked if this localization is affected by altering Rab11, using *1151GAL4* driver that specifically marks the AMP population (Figure 1). In controls, Rab11 is located in the cytoplasm and is observed as punctuate staining throughout the cytoplasm (Figure 9A–A’’). The AMPs in *1151GAL4;UAS-Rab11* ^*N124I*^ wing disc, showed complete loss of punctuate localization of Rab11. In these AMPs the protein was enriched at apical and sub-apical regions of the precursor cells instead of its typical cytoplasmic distribution observed in controls (Figure 9C–C’ D–D’).

**Figure 10:**
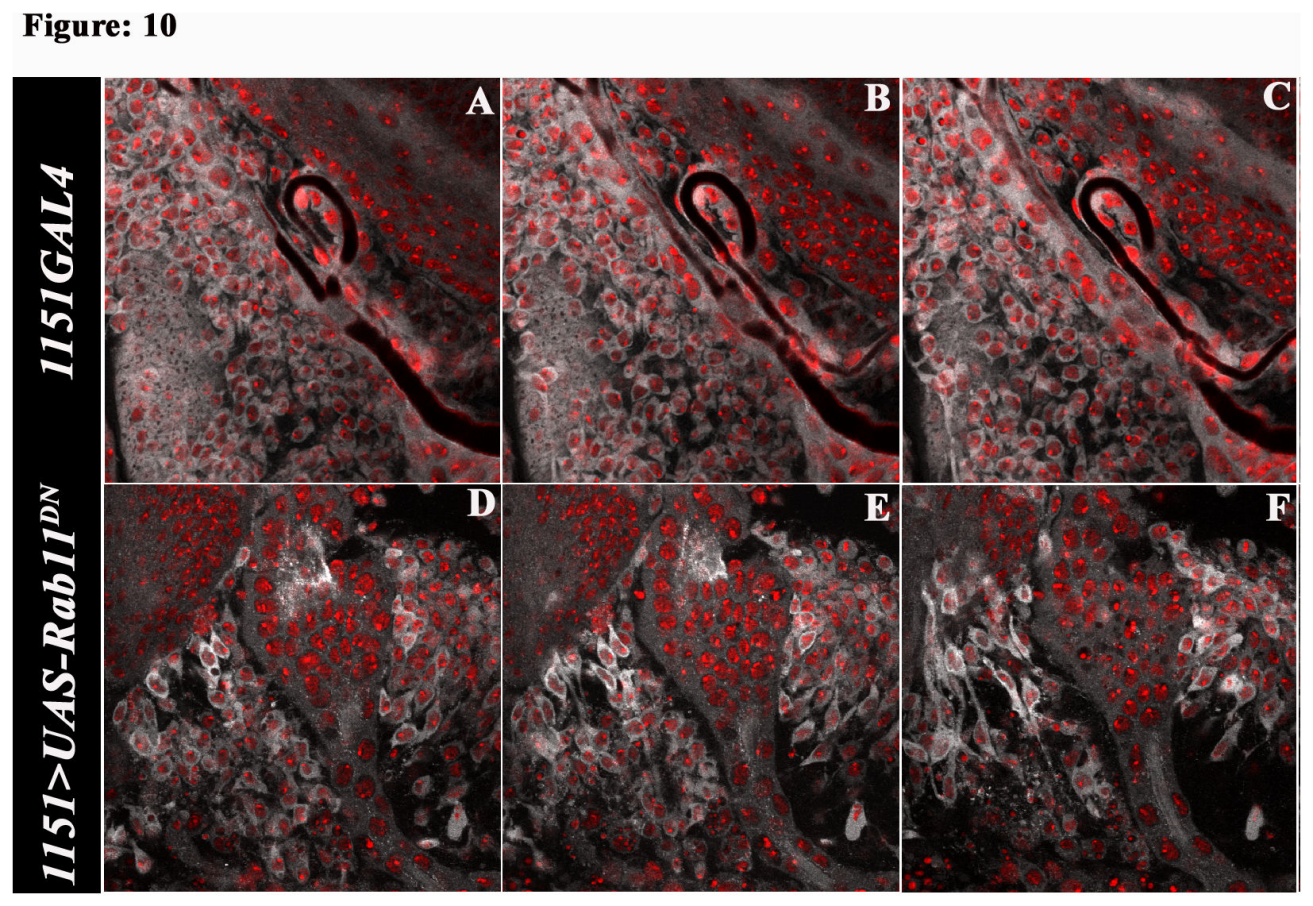
Myoblast acquire migratory characteristics on altering Rab11. Optical line sections of everting wing disc at 2.5h APF, at an interval of 0.75µm. The sections are displayed in the order of basal to apical polarity of the disc. Rab11 in white and DAPI in red. Pseudo colours have been used in both channels for better contrast; (A–C) Myoblast in the everting wing disc of controls (1151-GAL4/+) showing rounded morphology; (D–F) *1151*/*+; UAS-Rab11* ^*DN*^/*+*AMPs showing filopodial extensions at their polar ends along with increased Rab11 localization along the length of the filopodial extensions.

Intriguingly, we observed characteristic changes in the AMPs morphology at early stages of adult muscle development. The AMPs in *1151GAL4;UAS-Rab11* ^*N124I*^ wing disc were elongated and displayed a distinct pattern, where they were connected to the other AMPs of the population through long cellular protrusions. These AMPs showed loose, semi ordered contacts and were no longer seen adhering to each other (Figure 9C’’D’’). The myoblast in the basal layers of the wing disc were more severely affected and showed the characteristic spindle like morphology extending at the ends while the myoblast in the inner layers showed ‘tear drop’ morphology and loose contacts (Figure 9D–D”). In contrast, the AMPs in the control were closely associated and showed no protrusions at their polar ends. To observe fate of these AMPs at the time of wing disc eversion, we observed the AMPs at 2.5h APF. The myoblast in the everting disc showed long filopodial extensions at their apical ends. The everting discs in controls harboured myoblast that did not show filopodial extensions and were seen as rounded cells (Figure 10A–C). Thus, from these observations we surmise a functional requirement of Rab11 during early stages of adult muscle precursor differentiation.

## Discussion

The present study demonstrates the role of Rab11 in *Drosophila* adult myogenesis, by participating in the differentiation and growth of the adult muscle precursors. Blocking Rab11 function in the adult muscle precursors, which fuse to form the indirect flight muscles of the fly, results in rendering the flies completely flightless and non-viable. The IFM comprising of the differentially patterned DLMs and DVMs are affected to different extents. Abrogating normal Rab11 function or knocking down its function resulted in severely disrupted IFM structure. DLMs forming from larval templates [18] are reduced in number along with a significant reduction in their fibre size. On the other hand, the *de novo* developing DVMs are frequently absent. The DLMs in Rab11 hypomorphs are highly reduced, showing as a small constricted mass in one half of the thorax. Furthermore, we found that, Rab11 function is not only required for proper differentiation of muscles, but also essential for their growth during later half of muscle development, as on altering Rab11 in the indirect flight muscles results in degenerated muscles, broken fibres and large interfilament spaces. We surmise that the adult muscle phenotype may in part be due to the reduced proliferation of myoblast accompanied with cell shape changes. Thus, our data provide the first evidence of a trafficking protein playing an indispensable role in during the adult muscle development.

### Rab11 regulates proliferation of adult muscle precursors

The Indirect flight muscles are seeded from the adult muscle precursors that are formed in the early embryos [19]. During embryogenesis, a small population of myoblast that have persistent expression of Twist are set aside, which later function as precursors of the adult muscles. These Twist expressing cells actively proliferate during the larval instars to give rise to the adult muscles of the fly [18]. Our studies have shown that altering Rab11 in these proliferating myoblast significantly decreases their number during the extended period of myoblast proliferation. Delivery of new membranes and proteins is necessary to allow proper separation of the daughter cells, for which Rab11 activity is a prerequisite. Rab11 function during cytokinesis involves active membrane addition at the site of furrow formation during cytokinesis [29]. A similar role of Rab11 in mediating growth of lateral surfaces has been seen during cellularization in *Drosophila* embryo where it interacts with Nuclear fallout (Nuf) to speedup division [30,31]. Only recently, mouse homologue of human Rab11 family interacting protein 4, zRab11-FIP4 has been seen to regulate proliferation of retinal progenitors in Zebrafish embryos through Shh signalling [5]. In view of these it is most likely that over expressing the dominant negative Rab11 protein in the AMPs or knocking it down results in failure of separation of the two daughter cells, accounting for fewer number of AMPs, available at the time of fusion to form the adult muscle fibres.

### Rab11 is critical for differentiation of developmentally distinct DLMs and DVMs

Once the AMPs pool is generated by active proliferation, the next step is the accumulation of these myoblasts at the site of muscle formation, where the future DLMs and DVMs will be formed. The DLMs and DVMs, which constitute the thoracic Indirect Flight Muscles, have distinct modes of formation. The DLMs are formed using the larval oblique muscles (LOMs), which escape histolysis during metamorphosis and give rise to the future DLMs. These LOMs are supposed to function as founder cells, similar to the ones found in *Drosophila* embryo, and act as the fusion target for the myoblast, after which splitting of each template into two is activated during early hours of pupation. Splitting of each of these larval templates requires addition of new membrane that is delivered at the site of developing fibre to compensate for increased membrane surface of the adult fibre. The reduction of DLM fibres seen in majority of the *Rab11* ^*mo*^, *1151/+; UAS-Rab11* ^*N124I*^ */+* and *1151GAL4/+; UAS-Rab11* ^*RNAi*^ hemithoraces was probably due to failure of the larval templates to undergo splitting in absence of delivery of additional membrane during differentiation of these muscles, as the larval templates were found to be intact in these thoraces.

It has been shown from our lab that during embryonic myogenesis Rab11 is required for the fusion of myoblast and its depletion results in myoblast fusion defects accompanied with anomalies in the shape of the embryonic muscles [17]. It is hypothesized that proliferation must occur to restock the myoblast pool which is depleted as myoblast fuse to the larval templates. Thus, there is a feedback between fusion and proliferation to control the size of the myoblast pool, which during the period of peak fusion ensures that fibres of appropriate size are generated. We surmise that the reduced myoblast pool in the case of *1151/+; UAS-Rab11* ^*N124I*^ */+* and *1151GAL4/+; UAS-Rab11* ^*RNAi*^, along with dwindling fusion results in the formation of the thin fibres.

The DVMs that appear in three sets; DVM I consisting of 3 fibres, DVM II and DVM III with 2 fibres each, are formed using the dumbfounder positive founder cells. During adult myogenesis each individual muscle fibres of DVM bundle is seeded separately by a specific founder cell [21], in a fashion which is very similar to that in the *Drosophila* embryo, where a single embryonic founder cell is the precursor of a specific muscle fibre. Each of the fibre of a DVM bundle is formed using the ‘Founder Cell’ which is specified during development. The absence of individual fibres of DVM bundles is most likely due to the absence of the ‘Founder’ for the particular DVM fibre that failed to develop, at the site of muscle formation. Our studies have shown that Rab11 function is required for proper division of the muscle progenitors. It is most likely that failure of the progenitor to divide into individual founders for each of the DVM fibre results in the absence of formation of the respective DVM fibre of the bundles.

### Rab11 regulates early stages of AMPs differentiation and migration

Loss of Rab11 in *1151/+; UAS-Rab11* ^*N124I*^ */+* resulted in myoblast that showed ‘tear drop’ morphology and loss of cell-cell contacts. The AMPs in the wing disc were connected to each other through long cellular protrusions at their polar ends. Few hours later at the time of wing disc eversion the myoblast showed filopodial extensions at their apical ends which is a typical characteristic of migrating cells. The actin cytoskeleton in association with adherent junction helps in the adhesion process and maintenance of cell shape and morphology. The cell cycle exit is a prerequisite both for terminal differentiation and also to start the process of fusion [32]. Studies have demonstrated a key role for Rab11 in cytoskeleton organization [7] as disorganized actin cytoskeleton leads to degenerated rhabdomeres in adult *Drosophila* eye [10]. Disrupted cytoskeleton was also observed in embryos, testes and ovaries of Rab11 mutant individuals [33,34]. We show that AMPs fail to proliferate in the absence of Rab11 and prematurely exit the cell cycle. It is most likely that the premature cell cycle exit of the AMPs in *1151/+; UAS-Rab11* ^*N124I*^ */+* activates the differentiation machinery in the myoblast leading to acquisition of tear drop morphology of AMPs and migratory characteristics. Recently, Rab11 has been shown to regulate collective cell migration in *Drosophila* border cells [14] which further strengthens our proposed hypothesis. In view of these results along with demonstrated role of Rab11 in regulating cytoskeleton organisation it is most likely that loss of Rab11 results in acquisition of migratory characteristic of myoblast showing long extensions.

Taken together, the present study provides the first *in vivo* evidence for the requirement of a GTPase Rab11 in the differentiation and growth of the indirect flight muscles of *Drosophila* . Further studies are needed to understand the machinery that regulates the adult muscle development in *Drosophila.*

## Supporting Information

Figure S1 ***1151GAL4* driven UAS-GFP expression in muscle precursor cells of the wing imaginal disc** (A) wing disc notum showing GFP expression specifically in the AMPs (B) DAPI has been changed to red for better contrast (C) merged image.
(TIF)

Figure S2 **Altered Rab11 function in the indirect flight muscles during the period of growth results in thinning and degeneration.** (A) to (F) show representative examples of the DLM and DVM muscle phenotypes in Rab11 altered conditions; (A) *mhcF3-580-GAL4* flies showing six well organized DLMs; (B) *UAS-Rab11* ^*N124I*^ */+; mhcF3*-580-GAL4/+ hemithoraces showing thin unorganized and degenerated muscles. The DLMs occasionally show abnormal large gaps between two consecutive fibres. These gaps are absent in the controls muscles (C) *UAS-Rab11* ^*RNAi*^ */+; mhcF3*-580-GAL4/+ hemithoraces show significantly thin and abnormally spaced DLMs (D) *mhcF3-580-GAL4* fly hemithoraces showing DVM I,II and III (E) and (F) *UAS-Rab11* ^*N124I*^ */+; mhcF3*-580-GAL4/+ and *UAS-Rab11* ^*RNAi*^ */+; mhcF3-580-GAL4* /+ hemithoraces showing abnormally thin and degenerated DVMs.
(TIF)

Figure S3 **Loss of Rab11 in the AMPs does not induce apoptosis.** (A) Acridine orange stained wing disc notum of *1151-GAL4* third instar larvae showing absence of apoptotic cells (B) *1151/+; UAS-Rab11* ^*N124I*^ */+* and (C) *1151/+*; UAS*-Rab11* ^*RNAi*^ */+* wing disc did not show presence of any dead cells.
(TIF)

Video S1 (3GP)

## References

[1] Pfeffer S, Aivazian D (2004) Targeting Rab GTPases to distinct membrane compartments. Nat Rev Mol Cell Biol 5: 886-896. doi:10.1038/nrg1497. PubMed: 15520808. 15520808 10.1038/nrm1500

[2] Ali BR, Seabra MC (2005) Targeting of Rab GTPases to cellular membranes. Biochem Soc Trans 33: 652-656. doi:10.1042/BST0330652. PubMed: 16042566. 16042566 10.1042/BST0330652

[3] Chen W, Feng Y, Chen D, Wandinger-Ness A (1998) Rab11 is required for trans-Golgi network-to-plasma membrane transport and a preferential target for GDP dissociation inhibitor. Mol Biol Cell 9: 3241–3257. doi:10.1091/mbc.9.11.3241. PubMed: 9802909. 9802909 10.1091/mbc.9.11.3241 PMC25617

[4] Deretic D (1997) Rab proteins and post-Golgi trafficking of rhodopsin in photoreceptor cells. Electrophoresis 18: 2537–2541. doi:10.1002/elps.1150181408. PubMed: 9527482. 9527482 10.1002/elps.1150181408

[5] Muto A, Arai K, Watanabe S (2006) Rab11-FIP4 is predominantly expressed in neural tissues and involved in proliferation as well as in differentiation during zebrafish retinal development. Dev Biol 292: 90–102. doi:10.1016/j.ydbio.2005.12.050. PubMed: 16457799. 16457799 10.1016/j.ydbio.2005.12.050

[6] Jankovics F, Sinka R, Erdélyi M (2001) An interaction type of genetic screen reveals a role of the Rab11 gene in oskar mRNA localization in the developing Drosophila melanogaster oocyte. Genetics 158: 1177-1188. PubMed: 11454766. 11454766 10.1093/genetics/158.3.1177 PMC1461719

[7] Dollar G, Struckhoff E, Michaud J, Cohen RS (2002) Rab11 polarization of the *Drosophila* oocyte: a novel link between membrane trafficking, microtubule organization and oskar mRNA localization and translation. Development 129: 517–526. PubMed: 11807042. 11807042 10.1242/dev.129.2.517

[8] Satoh AK, O’Tousa JE, Ozaki K, Ready DF (2005) Rab11 mediates post-Golgi trafficking of rhodopsin to the photosensitive apical membrane of *Drosophila* photoreceptors. Development 132: 1487–1497. doi:10.1242/dev.01704. PubMed: 15728675. 15728675 10.1242/dev.01704

[9] Alone DP, Tiwari AK, Mandal L, Li M, Mechler BM et al. (2005) Rab11is required during *Drosophila* eye development. Int J Dev Biol 49: 873–879. doi:10.1387/ijdb.051986da. PubMed: 16172984. 16172984 10.1387/ijdb.051986da

[10] Tiwari AK, Roy JK (2009) Mutation in Rab11 results in abnormal organization of ommatidial cells and activation of JNK signalling in the *Drosophila* eye. Eur J Cell Biol 88: 445–460. doi:10.1016/j.ejcb.2009.02.188. PubMed: 19473727. 19473727 10.1016/j.ejcb.2009.02.188

[11] Lighthouse DV, Buszczak M, Spradling AC (2008) New components of the *Drosophila* fusome suggest it plays novel roles in signaling and transport. Dev Biol 317: 59–71. doi:10.1016/j.ydbio.2008.02.009. PubMed: 18355804. 18355804 10.1016/j.ydbio.2008.02.009 PMC2410214

[12] Bhuin T, Roy JK (2009) Rab11 is required for embryonic nervous system development fusion in *Drosophila* . Cell Tissue Res 335: 349–356. doi:10.1007/s00441-008-0711-8. PubMed: 19015884. 19015884 10.1007/s00441-008-0711-8

[13] Xu J, Lan L, Bogard N, Mattione C, Cohen RS (2012) Rab11 is Required for Epithelial Cell Viability, Terminal Differentiation, and Suppression of Tumor-Like Growth in the *Drosophila* Egg Chamber. PLOS ONE 6: e20180 PubMed: 21629779. 10.1371/journal.pone.0020180 PMC3100332 21629779

[14] Ramel D, Wang X, Laflamme C, Denise MJ, Emery G (2013) Rab11 regulates cell-cell communication during collective cell movements. Nat Cell Biol 15: 317-324. doi:10.1038/ncb2681. PubMed: 23376974. 23376974 10.1038/ncb2681 PMC4006229

[15] Bate M (1993) The mesoderm and its derivatives. In the Development of *Drosophila melanogaster* . Vol.2 (ed. M. Bate and A. Martinez-Arias), pp.1013-1090. New York: CSHL Press.

[16] Taylor MV (2006) Muscle Development in *Drosophila* . In: Sink, H. (Ed.), Comparison of muscle development in *Drosophila* and vertebrates. USA: Landes Bioscience.

[17] Bhuin T, Roy JK (2009) Rab11 is required for myoblast fusion in *Drosophila* . Cell Tissue Res 336: 489–499. doi:10.1007/s00441-009-0782-1. PubMed: 19370361. 19370361 10.1007/s00441-009-0782-1

[18] Fernandes J, Bate M, VijayRaghavan K (1991) Development of the indirect flight muscles of *Drosophila* . Development 113: 67–77. PubMed: 1765009. 1765009 10.1242/dev.113.1.67

[19] Bate M, Rushton E, Currie DA (1991) Cells with persistent twist expression are the embryonic precursors of adult muscles in *Drosophila* . Development 113: 79–89. PubMed: 1765010. 1765010 10.1242/dev.113.1.79

[20] Currie DA, Bate M (1991) Development of adult abdominal muscles in *Drosophila*: adult myoblasts express twi and are associated with nerves. Development 113: 91-102. PubMed: 1765011. 1765011 10.1242/dev.113.1.91

[21] Fernandes JJ, Keshishian H (2005) Motoneurons regulate myoblast proliferation and patterning in *Drosophila* . Dev Biol 277: 493–505. doi:10.1016/j.ydbio.2004.09.038. PubMed: 15617689. 15617689 10.1016/j.ydbio.2004.09.038

[22] Gaewski MK, Schulz AR (2010) CF2 Represses Actin 88F Gene Expression and Maintains Filament Balance during Indirect Flight Muscle Development in *Drosophila* . PLOS ONE 5: e10713. doi:10.1371/journal.pone.0010713. PubMed: 20520827. 20520827 10.1371/journal.pone.0010713 PMC2876027

[23] Abrams JM, White K, Fessler LI, Steller H (1993) Programmed cell death during *Drosophila* embryogenesis. Development 117: 29-43. PubMed: 8223253. 8223253 10.1242/dev.117.1.29

[24] Spreij TE (1971) Cell death during the development of the imaginal discs of *Calliphoraerythrocephala* . Neth J Zool 21: 221-264.

[25] Roy S, VijayRaghavan K (1997) Homeotic genes and the regulation of myoblast migration, fusion and fibre-specific gene expression during adult myogenesis in *Drosophila* . Development 124: 3333-3341. PubMed: 9310328. 9310328 10.1242/dev.124.17.3333

[26] Anant S, Roy S, VijayRaghavan K (1998) Twist and Notch negatively regulate adult muscle differentiation in *Drosophila* . Development 125: 136-1369. PubMed: 9502718. 10.1242/dev.125.8.1361 9502718

[27] McGuire SE, Mao Z, Davis RL (2004) Spatiotemporal gene expression targeting with the TARGET and gene-switch systems in Drosophila. Sci STKE 220: 16 PubMed: 14970377. 10.1126/stke.2202004pl6 14970377

[28] Lawrence PA, Brower DL (1982) Myoblasts from *Drosophila* wing discs can contribute to developing muscles throughout the fly. Nature 295: 55-57. doi:10.1038/295055a0.

[29] Wilson GM, Fielding AB, Simon GC, Yu X, Andrews PD, Hames RS Set al (2005) The FIP3-Rab11 protein complex regulates recycling endosome targeting to the cleavage furrow during late cytokinesis. Mol Biol Cell 16: 849-860 PubMed : 15601896 10.1091/mbc.E04-10-0927 PMC545916 15601896

[30] Pelissier A, Chauvin JP, Lecuit T (2003) Trafficking through Rab11endosomes is required for cellularization during *Drosophila* embryogenesis. Curr Biol 13: 1848–1857. doi:10.1016/j.cub.2003.10.023. PubMed: 14588240. 14588240 10.1016/j.cub.2003.10.023

[31] Riggs B, Rothwell W, Mische S, Hickson GR, Matheson J, Hays TS et al. (2003) Actin cytoskeleton remodelling duringearly *Drosophila* furrow formation requires recycling endosomal components nuclear-fallout and Rab11. J Cell Biol 163: 143–154. doi:10.1083/jcb.200305115. PubMed: 14530382. 14530382 10.1083/jcb.200305115 PMC2173427

[32] Zhang P, Wong C, Liu D, Finegold M, Harper JW et al. (1999) P21(CIP1) and p57 (KIP2) control muscle differentiation at the myogenin step. Genes Dev 13: 213-224. doi:10.1101/gad.13.2.213. PubMed: 9925645. 9925645 10.1101/gad.13.2.213 PMC316389

[33] Sasikumar S, Roy JK (2009) Developmental expression of Rab11, a small GTPbinding protein in *Drosophila* epithelia. Genesis 47: 32–39. doi:10.1002/dvg.20441. PubMed: 19039786. 19039786 10.1002/dvg.20441

[34] Tiwari AK, Alone DP, Roy JK (2008) Rab11 is essential for fertility in *Drosophila* . Cell Biol Int 32: 1158-1168. doi:10.1016/j.cellbi.2008.04.002. PubMed: 18640060. 18640060 10.1016/j.cellbi.2008.04.002

