# Supplementary figures and images for: Rab11 Plays an Indispensable Role in the Differentiation and Development of the Indirect Flight Muscles in Drosophila
Source: PLoS One. 2013 Sep 2;8(9):e73305. doi: 10.1371/journal.pone.0073305 (PMC3759402; doi:10.1371/journal.pone.0073305)

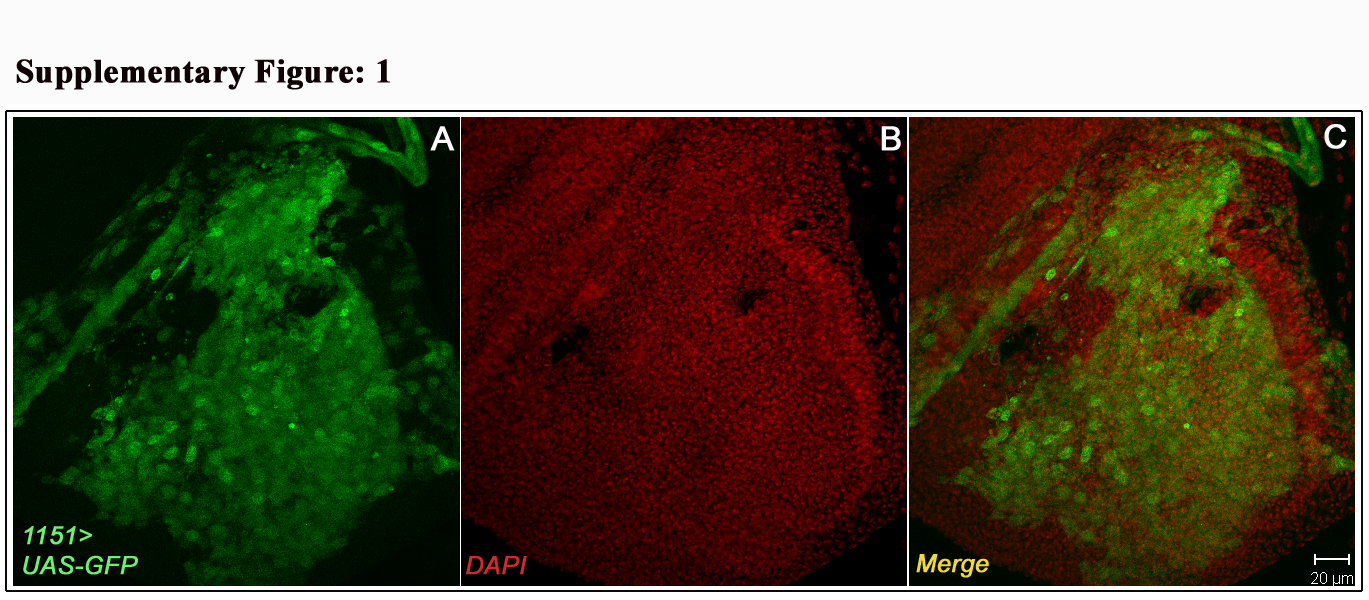

Supplement: Figure S1 — 1151GAL4 driven UAS-GFP expression in muscle precursor cells of the wing imaginal disc (A) wing disc notum showing GFP expression specifically in the AMPs (B) DAPI has been changed to red for better contrast (C) merged image. (TIF) [file pone.0073305.s001.tif]

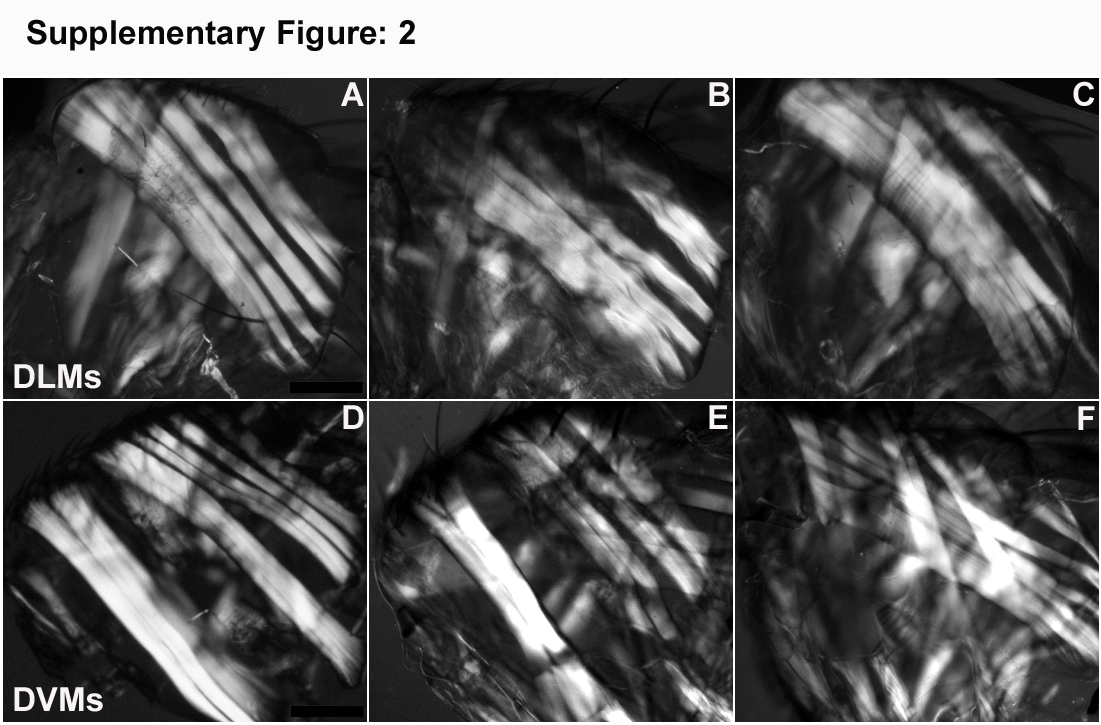

Supplement: Figure S2 — Altered Rab11 function in the indirect flight muscles during the period of growth results in thinning and degeneration. (A) to (F) show representative examples of the DLM and DVM muscle phenotypes in Rab11 altered conditions; (A) mhcF3-580-GAL4 flies showing six well organized DLMs; (B) UAS-Rab11 N124I /+; mhcF3-580-GAL4/+ hemithoraces showing thin unorganized and degenerated muscles. The DLMs occasionally show abnormal large gaps between two consecutive fibres. These gaps are absent in the controls muscles (C) UAS-Rab11 RNAi /+; mhcF3-580-GAL4/+ hemithoraces show significantly thin and abnormally spaced DLMs (D) mhcF3-580-GAL4 fly hemithoraces showing DVM I,II and III (E) and (F) UAS-Rab11 N124I /+; mhcF3-580-GAL4/+ and UAS-Rab11 RNAi /+; mhcF3-580-GAL4 /+ hemithoraces showing abnormally thin and degenerated DVMs. (TIF) [file pone.0073305.s002.tif]

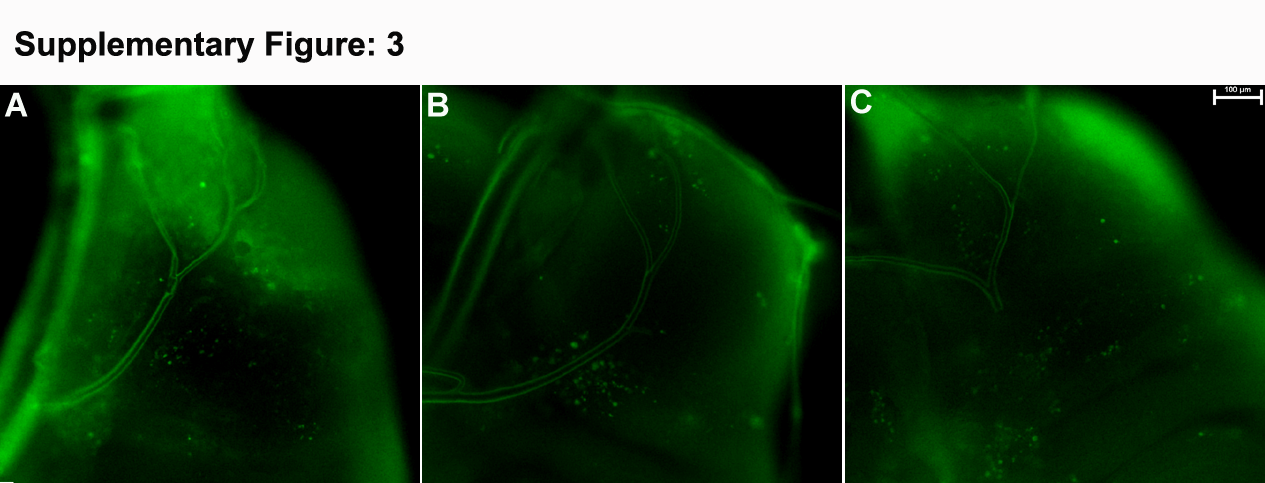

Supplement: Figure S3 — Loss of Rab11 in the AMPs does not induce apoptosis. (A) Acridine orange stained wing disc notum of 1151-GAL4 third instar larvae showing absence of apoptotic cells (B) 1151/+; UAS-Rab11 N124I /+ and (C) 1151/+; UAS-Rab11 RNAi /+ wing disc did not show presence of any dead cells. (TIF) [file pone.0073305.s003.tif]
